# Cancer Survivor Perspectives on Text Messages in a Technology-Based Physical Activity Intervention

**DOI:** 10.1093/geroni/igaa057.2241

**Published:** 2020-12-16

**Authors:** Nancy Gell, Christine Vatovec

**Affiliations:** 1 The University of Vermont, Burlington, Vermont, United States; 2 University of Vermont, Burlington, Vermont, United States

## Abstract

Messaging through mobile apps and texts are a common feature of technology-based physical activity interventions. We aimed to examine perspectives of mid-life and older cancer survivors on message content, timing, and two-way communication. We conducted qualitative interviews with 14 participants (Mean 59.9 years, Range 52-79) who completed a remotely delivered intervention that included text messages to support physical activity. After transcription, the interviews were coded and analyzed thematically using inductive, directed content analysis. Themes related to content preferences included personalization, accountability, perspective on the ‘bigger picture,’ and acknowledgement of PA-related achievements. Random timing for receipt was considered acceptable whereas interest in responding to messages was highly variable. Older cancer survivors’ preferences for highly personalized messages are an important consideration in designing technology-based physical activity interventions. A combination of accountability, relation to personal factors, and acknowledgement of goal attainment need to be considered in efforts to scale interventions. Part of a symposium sponsored by the Cancer and Aging Interest Group.

